# PPAR-γ Agonist GW1929 But Not Antagonist GW9662 Reduces TBBPA-Induced Neurotoxicity in Primary Neocortical Cells

**DOI:** 10.1007/s12640-013-9434-z

**Published:** 2013-10-17

**Authors:** Anna K. Wojtowicz, Konrad A. Szychowski, Małgorzata Kajta

**Affiliations:** 1 Laboratory of Genomics and Biotechnology, Animal Sciences Faculty, University of Agriculture, Redzina 1B, 30-248 Krakow, Poland; 2 Department of Experimental Neuroendocrinology, Institute of Pharmacology, Polish Academy of Sciences, Smetna 12, 31-343 Krakow, Poland

**Keywords:** TBBPA, PPAR-γ, Neurotoxicity, Apoptosis, Neocortex, GW1929

## Abstract

Tetrabromobisphenol A (2,2-bis(4-hydroxy-3,5-dibromophenyl)propane; TBBPA) is a widely used brominated flame retardant. TBBPA induces neuronal damage, but the mechanism by which this occurs is largely unknown. We studied the possible involvement of peroxisome proliferator-activated receptor gamma (PPAR-γ) in TBBPA-induced apoptosis and toxicity in mouse primary neuronal cell cultures. TBBPA enhanced both, caspase-3 activity and lactate dehydrogenase (LDH) release in neocortical cells after 6 and 24 h of exposition. These data were supported at the cellular level with Hoechst 33342 staining. Immunoblot analyses showed that, compared with control cells, 10 μM TBBPA decreased the expression of PPAR-γ protein in neocortical neurons after 1–24 h of exposure. Co-treatment with TBBPA and GW1929 inhibited the TBBPA-induced caspase-3 activity, apoptotic body formation, and LDH release as well as TBBPA-induced decrease in PPAR-γ protein expression. Thus, our data support neuroprotective potential of PPAR-γ agonists. The PPAR-γ antagonist GW9662 prevented the TBBPA-induced decrease in PPAR-γ protein level, but it potentiated TBBPA-induced apoptotic and neurotoxic effects, which suggest that the mechanism of TBBPA action in neuronal cells is not only PPAR-γ-dependent. Therefore, further studies of the mechanism of TBBPA action in the nervous system are needed.

## Introduction

Tetrabromobisphenol A (2,2-bis(4-hydroxy-3,5-dibromophenyl)propane; TBBPA) is a widely used brominated flame retardant (BFR). TBBPA is used as a replacement for the polybrominated diphenylethers (PBDEs), which are persistent environmental BFRs that have documented negative effects on human health (Talsness et al. 2009; Kiciński et al. 2012). TBBPA can be utilized both as a reactive flame retardant in epoxy resin-printed circuit boards and as an additive flame retardant in a wide variety of commercial and household products, such as plastics, textiles and electronic appliances, including computers and televisions (Alaee et al. 2003; Covaci et al. 2009; de Wit et al. 2010). This versatility has resulted in a dramatic increase in TBBPA production. Several studies have shown that when this component is used as an additive in polymers, it can be easily released from the treated products (Sellstrom and Jansson 1995; Birnbaum and Staskal 2004). Despite its short half-life, TBBPA may accumulate in tissues following repeated exposure (Sjödin et al. 2003). Although many studies indicate that rats and humans quickly metabolize TBBPA due to its rapid conjugation with glucuronic acid and elimination in the bile, TBBPA has been detected in cow and human milk, serum, adipose tissue and umbilical cord serum (Thomsen et al. 2002; Johnson-Restrepo et al. 2008). In human serum samples 0.24–0.71 ng of TBBPA/g lipid were reported which is equal to 1.8–5.3 pM, using a conversion factor of 400 mg lipid/100 ml serum (Thomsen et al. 2002). Although the half-life of TBBPA is about 2 days in humans, continuous uptake of this compound may increase its concentrations and potentiate toxicological effects of TBBPA (Sjödin et al. 2003). According to toxicokinetic study of Schauer et al. (2006), a single oral dose of 300 mg/kg TBBPA to adult humans resulted in a plasma concentration of over 100 μM within 3 h of exposure, and this level was maintained for approximately 6 h (elimination half-life of 13 h). Therefore, living organisms may be temporally exposed to even higher concentrations of TBBPA than they have been reported in plasma or urine samples. As numerous studies have demonstrated, TBBPA causes pathological changes in many organs, particularly the thyroid and liver, and it negatively affects the immune and reproductive systems (Van der Ven et al. 2008; Kibakaya et al. 2009). TBBPA also has carcinogenic properties, which means that exposure to this compound may lead to the development of numerous types of cancers (Shi et al. 2010). In addition, TBBPA accumulates in different brain regions and induces behavioral alterations (Nakajima et al. 2009; Viberg and Eriksson 2011). However, only a few studies have investigated the mechanism through which TBBPA acts. TBBPA was recently shown act as a peroxisome proliferator-activated receptor gamma (PPAR-γ) ligand in NIH3T3-L1 cells (Riu et al. 2011a).

PPAR-γ is a ligand-activated transcription factor that belongs to the nuclear receptor superfamily. It regulates the expression of genes that are related to metabolic processes and reducing inflammation. Importantly, PPAR-γ is widely expressed in the brain, where it has a crucial role in the regulation of nervous cell proliferation, differentiation, and apoptosis. PPAR-γ has a protective function in neurons. Yi et al. (2009) showed that PPAR-γ activation confers neuroprotection through anti-inflammatory, anti-apoptotic, and anti-oxidative mechanisms. In addition, the activation of PPAR-γ receptor ameliorates neurodegenerative diseases (Kitamura et al. 1999; Heneka et al. 2000; Garrido-Gil et al. 2012). Although PPAR-γ activation reduces brain tissue damage in experimental models of brain diseases (Pereira et al. 2006; Tureyen et al. 2007; Zhao et al. 2011; Ridder and Schwaninger 2012), how PPAR-γ activity is regulated in neurons is unclear.

The aim of this study was to investigate the effect of TBBPA on the viability and apoptosis of mouse neocortical neurons at 7 days in vitro (DIV) after 6 and 24 h of exposure. To explore the mechanism of TBBPA action on neocortical cells, we studied the involvement of PPAR-γ in TBBPA-induced neurotoxicity.

## Materials and Methods

### Reagents

Neurobasal medium without phenol red and B27 supplement were purchased from Life Technologies. Trypsin, charcoal/dextran-treated fetal bovine serum, penicillin, streptomycin, TRIS, HEPES, CHAPS, DTT, EDTA, Tween 20, bromophenol blue, staurosporine, TBBPA, and DMSO were purchased from Sigma-Aldrich (St. Louis, MO, USA). The PPAR-γ agonist GW1929 and the PPAR-γ antagonist GW9662 were purchased from Sigma-Aldrich. Stock solutions of these test compounds were prepared in DMSO and were added to Neurobasal medium. The final concentration of DMSO in the culture medium was always 0.1 %. The cytotoxicity lactate dehydrogenase (LDH) detection kit was purchased from Roche Applied Science (Germany).

### Primary Neocortical Cell Cultures

The experiments were performed on primary cultures of mouse cortical neurons. The primary cultures of neocortical neurons were prepared from the fetuses of pregnant female Swiss mice as previously described in detail (Brewer 1995; Kajta et al. 2005). Brain tissues were collected from mouse embryos on days 15 or 16 of gestation. Pregnant females were anesthetized with CO_2_ vapor and killed by cervical dislocation. Animal care followed official governmental guidelines, and all efforts were made to minimize the number of animals used and their suffering. All procedures were performed in accordance with the National Institutes of Health Guidelines for the Care and Use of Laboratory Animals and were approved by the Bioethics Commission, as compliant with Polish law.

Brains were removed from fetuses and the cortical tissues were dissected. The dissected tissue was minced into small pieces and then gently digested with trypsin and DNAse I. Then, the cells were centrifuged, and the pellet was resuspended in phenol red-free Neurobasal medium (Life Technologies) supplemented with 5 % fetal calf serum. Cells were plated onto poly-l-ornithine (0.01 mg per ml)-coated multi-well plates. After 2 days, the culture medium was exchanged to Neurobasal medium supplemented with B27 (2 μL/mL), glutamine (2 mM), 50 U/mL penicillin, and 0.05 mg/mL streptomycin, which is recommended for primary neuronal cultures (Brewer 1995; Kajta et al. 2005). This procedure typically yields cultures that contain about 90 % neurons and 10 % astrocytes (Kajta et al., 2004). The cultures were maintained at 37 °C in a humidified atmosphere containing 5 % CO_2_ and were cultivated for 7 days in vitro prior to the experiment. The culture medium was changed prior to treating cultures with all compounds selected for this study. Then, primary neocortical cell cultures were exposed to experimental doses of TBBPA for 6 h or 24 h.

### Treatment

For the experiments, the cells were plated in 96-well plates at a density 2 × 10^5^ cells per cm^2^ and cultured in the presence of TBBPA, in a concentrations range from 1 nM to 100 μM TBBPA. TBBPA was dissolved in DMSO, resulting in a final vehicle concentration of 0.1 % (v/v). Control (no vehicle) and DMSO-treated wells were included in the experimental design to determine the effect of DMSO (result not shown). To study whether PPAR-γ is involved in the neurotoxic effect of TBBPA, cells were co-treated with 10 μM TBBPA and 10 μM GW1929 or GW9662. After 6 or 24 h of culture, 100 μl medium was collected for the LDH analysis, and the cells were collected and frozen at −70 °C for the caspase-3 activity measurements.

### LDH Cytotoxicity Assay

The cytotoxicity detection kit (Roche Applied Science, Germany) is a colorimetric assay for the quantification of cell death and cell lysis based on the LDH activity released from the cytosol of damaged cells into the supernatant. An increase in the amount of dead or plasma membrane-damaged cells results in an increase in the LDH activity in the culture supernatant. After 6 or 24 h of treatment with the rising concentrations of TBBPA, 100 μl of the culture supernatants was collected and incubated with the reaction mixture from the kits. After 30 min, the reaction was stopped by adding 1 N HCl, and the absorbance was measured at a wavelength of 490 nm with a reference wavelength of 600 nm in the microELISA plate reader Bio-Tek Instruments (Biokom).

### Caspase-3 Activity

Caspase-3 activity was used as a marker for cell apoptosis and was assessed according to Nicholson et al. (1995). Cultured neurons were lysed with a lysis buffer (50 mM HEPES, pH 7.4, 100 mM NaCl, 0.1 % CHAPS, 1 mM EDTA, 10 % glycerol, 10 mM DTT). Lysates were incubated with the caspase-3 substrate Ac-DEVD-pNA (*N*-acetyl-Asp-Glu-Val-Asp-p-nitroanilide; Sigma-Aldrich) at 37 °C. Cells treated with 1 μM staurosporine were used as a positive control. After 30 min, the absorbance of the lysates was measured at 405 nm in a microplate reader (Bio-Tek ELx800). The amount of colorimetric product was monitored continuously for 120 min. Data were analyzed with KCJunior (Bio-Tek Instruments) and normalized to the absorbance in vehicle-treated cells. The results are expressed as the mean % of control from eight separate samples ±SEM, and samples were run in quadruplicate.

### Western Blot Analysis

For immunoblotting, the cells were lysed in ice-cold lysis buffer containing 50 mM HEPES, 100 mM NaCl, 0.1 % CHAPS, 1 mM EDTA, 10 % glycerol, and 10 mM DTT. Then, the lysates were sonicated and clarified by centrifugation at 15.000×*g* at 4 °C for 30 min. The protein concentrations in the supernatants were determinate with the Bradford reagent (BioRad Protein Assay; BioRad Laboratories, Munchen, Germany) using bovine serum albumin (BSA) as the standard. From the whole explant lysate, 100 μg of total protein was reconstituted in the appropriate amount of sample buffer, consisting of 125 mM Tris, pH 6.8, 4 % SDS, 25 % glycerol, 4 mM EDTA, 20 mM DTT, and 0.01 % bromophenol blue. Samples were separated by 7.5 % SDS–polyacrylamide gel electrophoresis in a Bio-Rad Mini-Protean II Electrophoresis Cell, and the proteins were then transferred to nitrocellulose membranes using a Bio-Rad Mini Trans-Blot apparatus. Following the transfer, the membranes were washed, and nonspecific binding sites were blocked with 5 % dried milk and 0.2 % Tween 20 in 0.02 M TBS for 2 h. Then, the membranes were incubated overnight with the PPAR-γ receptor antibody (goat anti-human polyclonal antibody, Santa Cruz Biotechnology, Inc.) diluted at 1:100 in TBS/Tween at 4 °C. After incubation with the primary antibody, the membranes were washed with TBS and 0.02 % Tween 20 and incubated for 2 h with horseradish peroxidase-conjugated secondary antibody (donkey anti-goat IgG, Santa Cruz Biotechnology, Inc.) diluted at 1:500 in TBS/Tween. To control for the amount of protein that was loaded onto the gel, the membranes were stripped and reprobed with an anti-β-actin antibody (Sigma-Aldrich). Signals were detected by chemiluminescence (ECL) using a Western Blotting Luminol Reagent (Santa Cruz Biotechnology, Inc.) and visualized with the use of a Syngene GBOX and GeneSnap software.

### Identification of Apoptotic Cells with Hoechst 33342 Staining

Apoptotic cells exhibit nuclear condensation and DNA fragmentation, which can be detected by vital staining with Hoechst 33342 (Sigma-Aldrich). For this purpose, neurons were seeded on polyornithine-coated coverslips placed in 24-well plates at a density of 2.5 × 10^5^/well and were initially cultured for 7 days to allow for differentiation. Then, the medium was changed to Neurobasal supplemented with B27 in the presence of 10 μM of TBBPA, and the cells were cultured for an additional 24 h. After this period, the cells were washed with PBS and exposed to Hoechst 33342. Hoechst 33342 was diluted with PBS and added to the medium at a final concentration of 10 μM. Cells were incubated for 15 min in an atmosphere of 5 %CO_2_/95 % air at 37 °C and then visualized with a fluorescent microscope (Nikon, Japan).

### Statistical Analysis

Data are presented as the mean ± SEM of four independent experiments. Each treatment was repeated eight times (*n* = 8) and run in quadruplicate; thus, the total number of replicates was 32. The average of the quadruplicate samples was used for the statistical calculations. Data were analyzed by one-way analysis of variance (ANOVA) followed by Tukey’s multiple comparison procedure.

## Results

### Effects of TBBPA on Caspase-3 Activity in Neocortical Primary Cell Cultures (7 DIV)

**Fig. 1:**
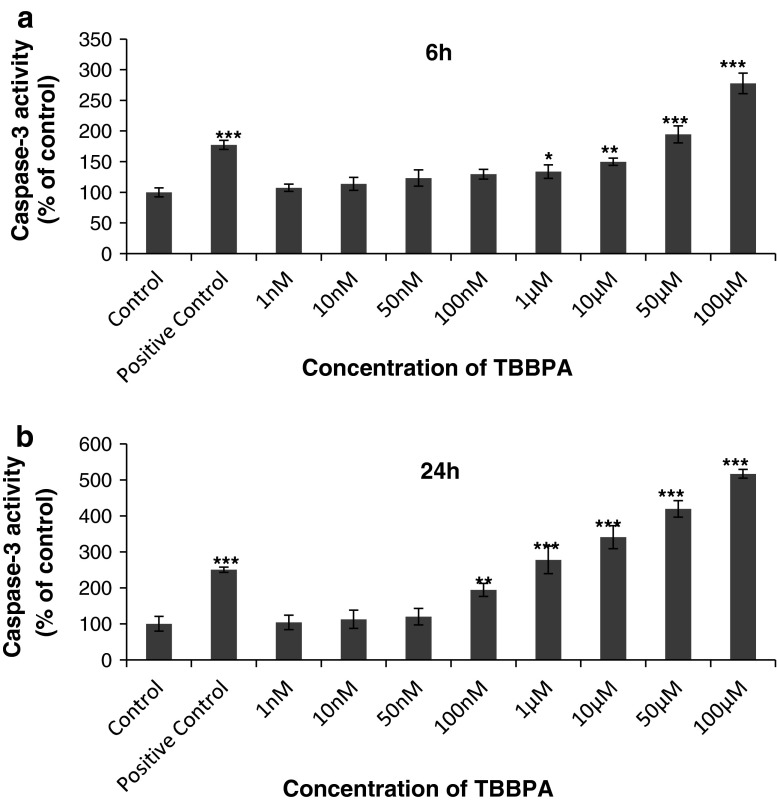
The effect of increasing concentrations of TBBPA (1, 10, 50, and 100 nM and 1, 10, 50, and 100 μM) on caspase-3 activity in cultured neocortical neurons cells after 6 **a** and 24 **b** h of exposure. Cell treated with 1 μM of staurosporine were used as a positive control. Each point represents the mean ± SEM of four independent experiments, each of which consists of eight replicates per treatment group. ***p < 0.001, **p < 0.01, and *p < 0.05 versus the control cultures

Caspase-3 activity significantly increased following TBBPA exposition for 6 h at doses of 1, 10, 50, and 100 μM of TBBPA compared with the vehicle control (Fig. 1a). These concentrations of TBBPA increased caspase-3 activity compared with the vehicle control (33.6, 49.8, 84.4, and 165.8 %, respectively). After 24 h of exposure, the increase in caspase-3 activity was dose-dependent, starting from the 100 nM concentration. The caspase-3 activity induced by the exposure to 100 nM and 1, 10, 50 and 100 μM TBBPA increased compared with the vehicle control (94.4, 177.7, 240.7, 319.4, and 416.7 %, respectively) (Fig. 1b).

### Effects of TBBPA on LDH Release in Neocortical Primary Cell Cultures (7 DIV)

**Fig. 2:**
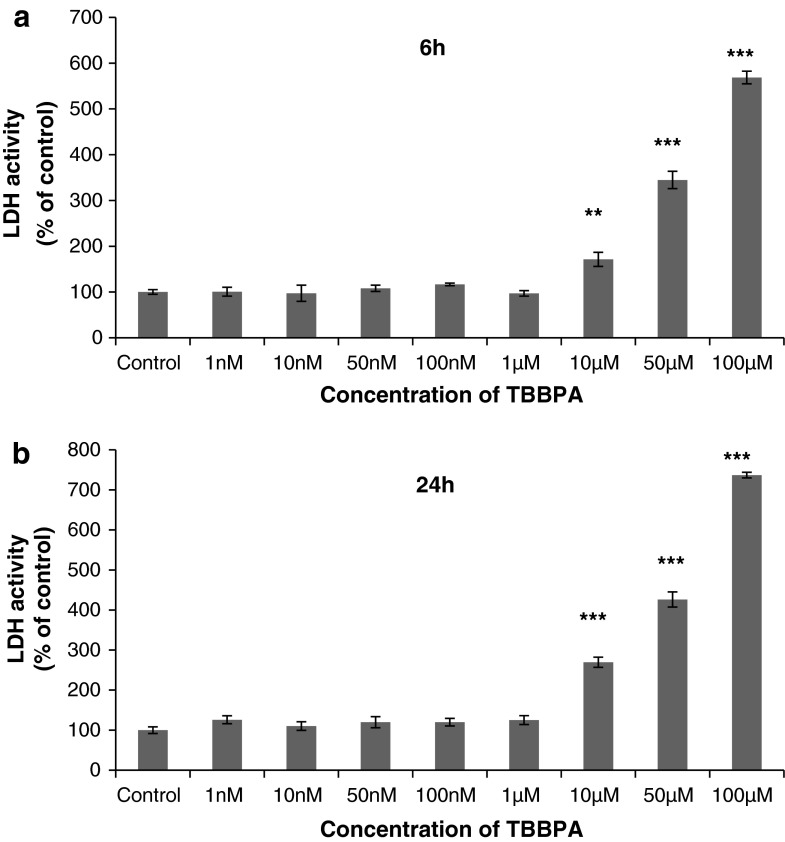
The effect of increasing concentrations of TBBPA (1, 10, 50, and 100 nM and 1, 10, 50, and 100 μM) on LDH activity in cultured neocortical neurons cells after 6 **a** and 24 **b** h of exposure. Data are the mean ± SEM of four independent experiments, each of which consists of eight replicates per treatment group. ***p < 0.001 versus the control cultures

In neocortical cell cultures, after 6 and 24 h of exposure to 10, 50, or 100 μM TBBPA, LDH activity was increased compared with the vehicle control (71.4, 244.9, and 468.6 %, respectively, after 6 h; 169.4, 326.2, and 636.9 %, respectively, after 24 h) (Fig. 2a, b).

### Effect of TBBPA on the Expression of PPAR-γ in Neocortical Cell Cultures

**Fig. 3:**
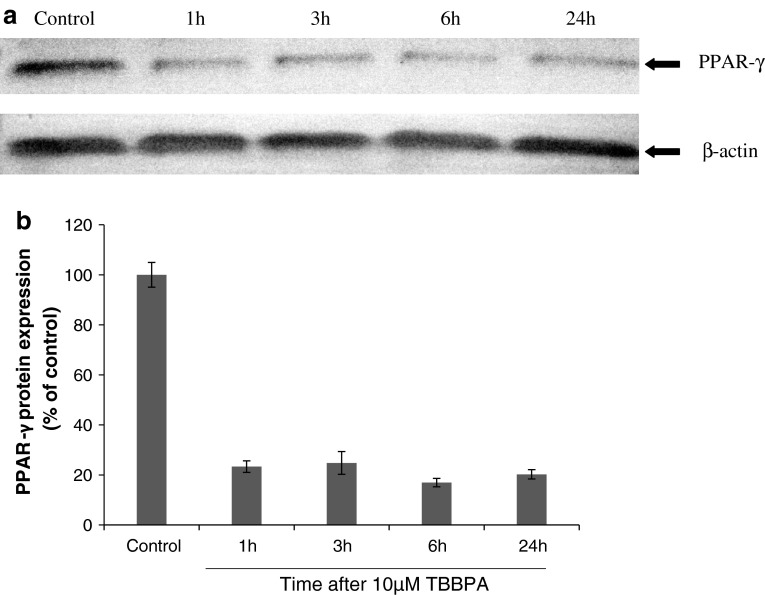
Representative Western blot of PPAR-γ protein levels in neocortical neurons treated with 10 μM of TBBPA for 0, 1, 3, 6, and 24 h (**a**). PPAR-γ bands were quantified by densitometry. The results are shown as the percentage of PPAR-γ protein relative to the control. Each column represents the mean ± SEM of three independent experiments (**b**). The blots were stripped and reprobed with anti-β-actin antibody to control for the amounts of protein loaded onto the gel

**Fig. 4:**
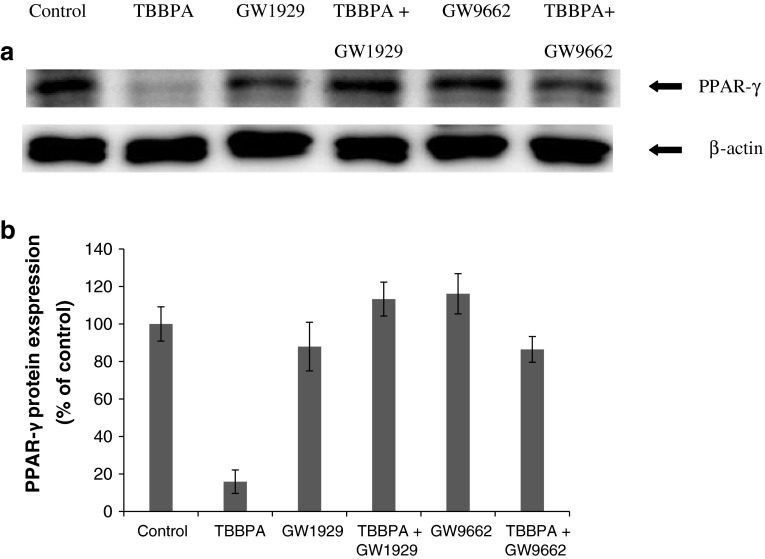
Representative Western blot of PPAR-γ protein levels in neocortical neurons treated with TBBPA (10 μM); GW1929 (10 μM); cells co-treated with GW1929 (10 μM) and TBBPA (10 μM); GW9662 (10 μM); cells co-treated with GW9662 (10 μM) and TBBPA (10 μM) (**a**). PPAR-γ bands were quantified by densitometry. The results are shown as the percentage of PPAR-γ protein relative to the control. Each column represents the mean ± SEM of three independent experiments (**b**). The blots were stripped and reprobed with anti-β-actin antibody to control for the amounts of protein loaded onto the gel

Immunoblot analyses showed that, compared with control cells, 10 μM of TBBPA decreased the level of PPAR-γ protein in neocortical neurons after 1–24 h of exposure (Fig. 3a, b). PPAR-γ agonist and antagonist alone as well as in the presence of TBBPA did not affect the PPAR-γ protein level (Fig. 4a, b). All samples had equal protein concentrations, as verified based on the expression of β-actin (loading control).

### Effects of the PPAR-γ Agonist on TBBPA-Stimulated Caspase-3 Activity and TBBPA LDH Release in Neocortical Cell Cultures

**Fig. 5:**
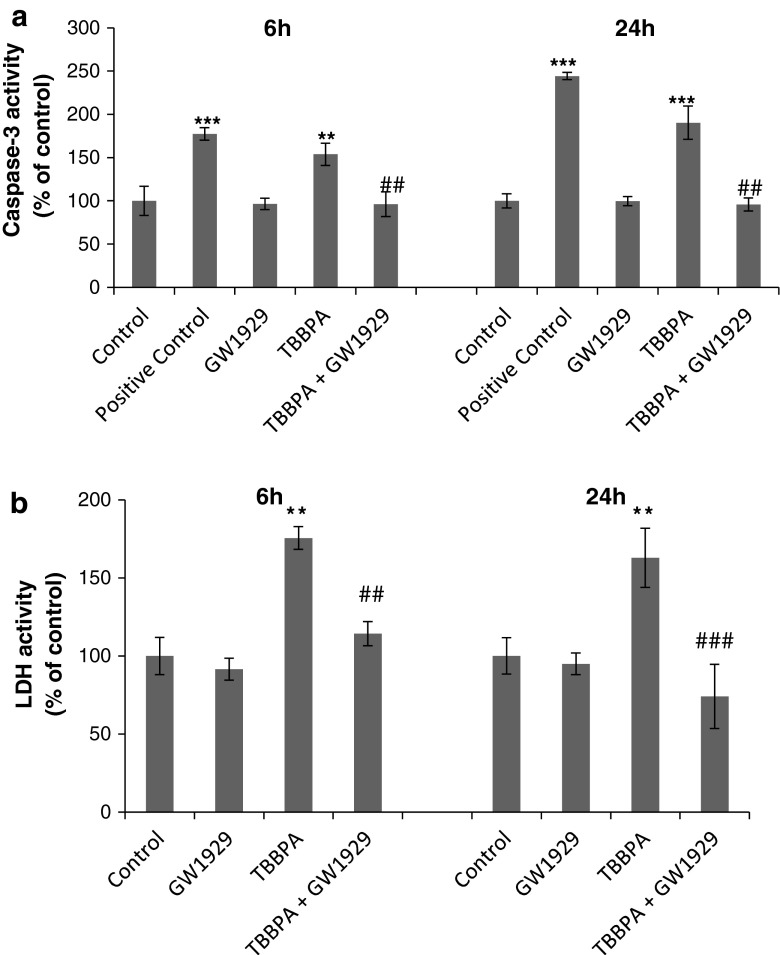
The effect of 10 μM of TBBPA on the caspase-3 **a** and LDH **b** activity in the presence of PPAR-γ agonist GW1929 in neocortical neuron cultures after 6 and 24 h of exposure. Cell treated with 1 μM of staurosporine were used as a positive control. Data are the mean ± SEM of four independent experiments, each of which consists of eight replicates per treatment group. ***p < 0.001 and **p < 0.01 versus the control group; ^###^ *p* < 0.001 and ^##^ *p* < 0.01 versus the TBBPA-stimulated group

In the presence of PPAR-γ agonist GW1929, the TBBPA-induced caspase-3 increase was diminished. Pre-treating neuron cultures with GW1929 inhibited caspase-3 activity, compared with TBBPA treatment, by 57.7 and 94.5 % after 6 and 24 h of exposure, respectively (Fig. 5a). The cytotoxic effect of 10 μM TBBPA, measured based on LDH release, in the presence of GW1929 was also inhibited after 6 and 24 h of exposure to 51.3 and 90.8 %, respectively, compared with the TBBPA-stimulated LDH release (Fig. 5b).

### Effects of the PPAR-γ Antagonist on TBBPA-Stimulated Caspase-3 Activity and TBBPA LDH Release in Neocortical Cell Cultures

**Fig. 6:**
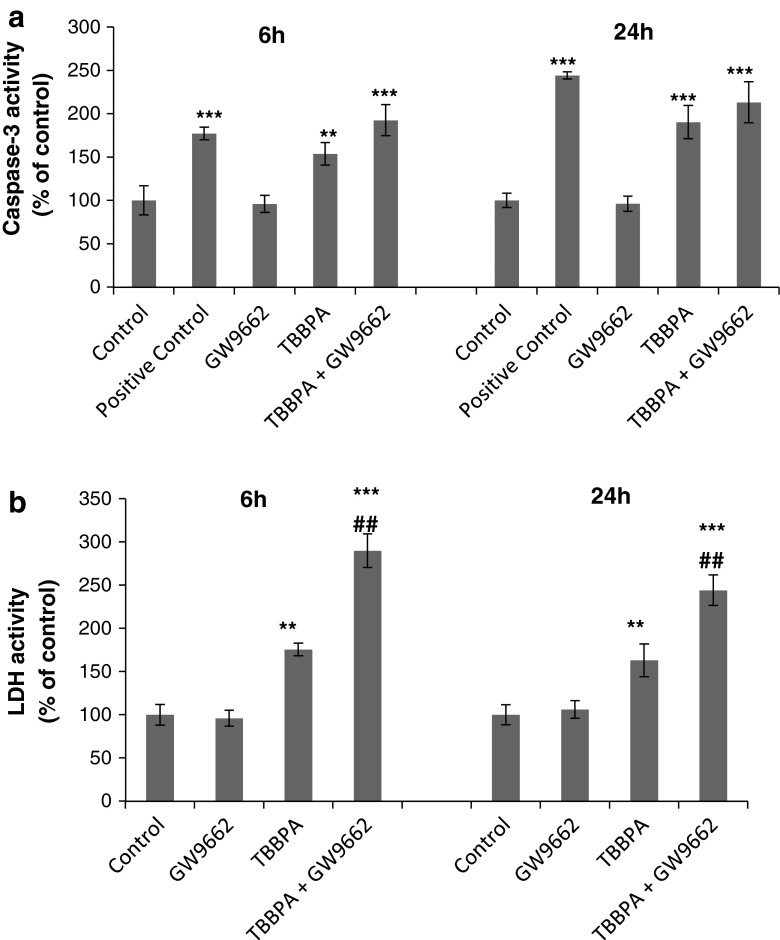
Effect of 10 μM of TBBPA on caspase-3 **a** and LDH **b** activity in the presence of PPAR-γ antagonist GW 9662 in neocortical neuron cultures after 6 and 24 h of exposure. Cell treated with 1 μM of staurosporine were used as a positive control. Data are the mean ± SEM of four independent experiments, each of which consists of eight replicates per treatment group. ***p < 0.001 and **p < 0.01 versus the control group; ^##^ *p* < 0.01 versus the TBBPA-stimulated group

The opposite effect was observed when cells were co-treated with PPAR-γ antagonist GW9662 and 10 μM TBBPA. After 6 and 24 h of treatment, caspase-3 activity was 38.7 and 22.8 %, respectively (Fig. 6a). Pretreating cells with GW9662 enhanced the cytotoxic effect of TBBPA, and LDH release was increased by 114.2 and 81.2 % after 6 and 24 h of exposition, respectively, compared with the TBBPA-stimulated LDH release (Fig. 6b).

### Effect of TBBPA on Hoechst 33342 Staining in Neocortical Cell Cultures

**Fig. 7:**
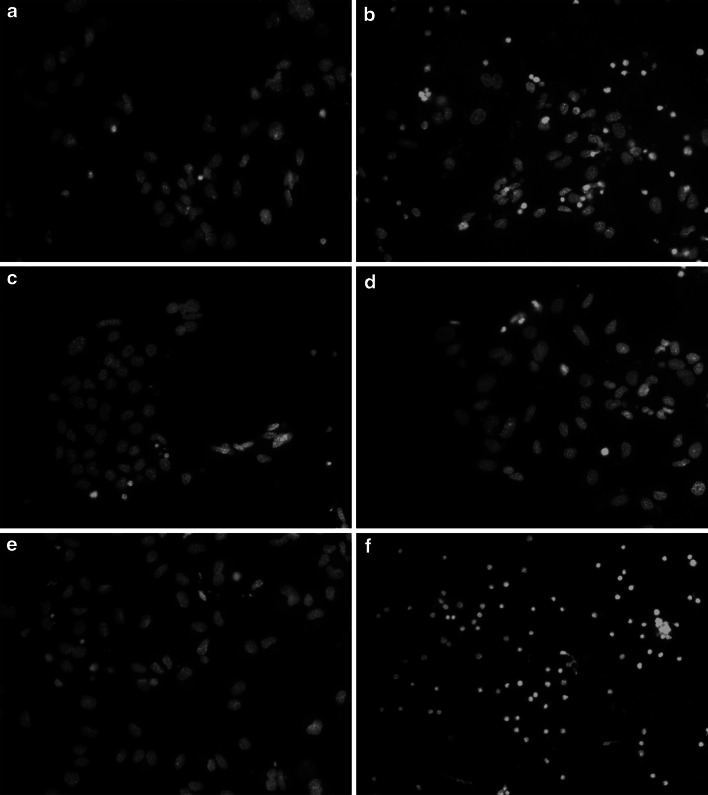
The effect of TBBPA on Hoechst 33342 staining in neocortical neuron cell cultures, examined 24 h post treatment, **a** control cells; **b** TBBPA-treated cells (10 μM); **c** GW1929-treated cells; **d** cells co-treated with GW1929 (10 μM) and TBBPA (10 μM); **e** GW9662-treated cells; **f** cells co-treated with GW9662 (10 μM) and TBBPA (10 μM). Cells with light-colored cytoplasm were identified as living cells. Cells with bright fragmented nuclei showing condensed chromatin were identified as undergoing apoptosis. Photomicrographs are shown in ×200 magnification

To confirm that apoptosis was induced, the neurocortical neurons were stained with Hoechst 33342 to show DNA fragmentation. Apoptotic bodies appeared as bright blue fragmented nuclei that showed condensed chromatin, which is typical of apoptotic cells. In the control culture, healthy cells with intact nuclei and diffuse fluorescence were mostly observed (Fig. 7a). The apoptotic bodies were observed in cells after 24 h of exposure to 10 μM TBBPA (Fig. 7b) or to co-treatment with GW9662 and 10 μM of TBBPA (Fig. 7f). In cells cultured in the presence of the PPAR-γ agonist GW1929 and 10 μM TBBPA (Fig. 7d), we observed only a few apoptotic bodies, as observed in untreated control cells.

## Discussion

This study demonstrated that micromolar concentrations of TBBPA treatment has a cytotoxic effect, indicated by LDH release in mouse neocortical cell cultures, after as little as 6 h of incubation. Our data also showed that TBBPA could induce caspase-3 activity and apoptotic body formation in these cells. Furthermore, the apoptotic effects were induced by lower concentrations of TBBPA than were required to induce LDH release. These results suggest that in primary neocortical cell cultures, TBBPA induces cell death by caspase-3-dependent apoptosis, and then the cells undergo secondary necrosis. Similar results were previously obtained by Reistad et al. (2007), who showed that micromolar concentrations of TBBPA-induced apoptotic body formation and DNA fragmentation followed by the cerebellar granule cell death. In contrast to our study, these authors did not detect activation of caspase-3, and suggested a caspase-independent mechanism of TBBBA-induced apoptotic cell death. Interestingly, TBBPA-induced caspase-3-dependent apoptosis was observed in rat pheochromocytoma PC-12 and SH-SY5Y human neuroblastoma cells (Al-Mousa and Michelangeli 2012; Hendriks et al. 2012). In addition to above mentioned apoptotic effects, TBBPA may increase intracellular calcium level, induce reactive oxygen species formation, as well as to activate microglia and cytokine synthesis.

Recent studies on TBBPA affinity to nuclear receptors showed that intracellular actions of TBBPA are mediated by PPAR-γ (Riu et al. 2011a, b). The ability of TBBPA to activate PPAR-γ receptors were demonstrated in reported cell lines (HeLa cells), zebrafish, and Xenopus. These authors, however, studied only TBBPA-binding affinity to PPAR-γ, but not TBBPA-induced apoptosis and toxicity. Therefore, we hypothesize that in our study, PPAR-γ is involved in apoptotic effects of TBBPA in neuronal cells. We demonstrated that PPAR-γ protein is expressed in mouse neocortical cell cultures. The presence of PPAR-γ has previously been documented in various areas of the adult brain and spinal cord and the highest level of PPAR-γ expression was observed in human and mouse neural stem cells (Cristiano et al. 2001; Moreno et al. 2004; Cimini et al. 2005; Wada et al. 2006; Chiang et al. 2013). It has been postulated that in the nervous system PPAR-γ regulates the apoptosis and neurotoxicity (Kim et al. 2003; Akasaki et al. 2006; Gray et al. 2012; Zeng et al. 2012). The recent study showed that activation of PPAR-γ down-regulated caspase-3 activity and viability of human neuronal stem cells, and protected murine cortical neurons against ischemia (Kaundal and Sharma 2011; Zeng et al. 2012; Chiang et al. 2013). However, the mechanism underlying the cytotoxic and pro-apoptotic effects of TBBPA is still unclear.

Our study demonstrated that TBBPA-induced apoptosis in neuronal cells which was accompanied by reduction of PPAR-γ protein expression, much below the control level. Treatment with PPAR-γ agonist GW1929 did not change control levels of any measured parameter. Co-treatment with TBBPA and GW1929 inhibited the TBBPA-induced caspase-3 activity, apoptotic body formation, and LDH release as well as TBBPA-induced decrease in PPAR-γ protein expression. Our data support neuroprotective potential of PPAR-γ agonists. The protective effects of PPAR-γ agonists have been shown in many experimental models of brain injury, including cerebral ischemia (Shimazu et al. 2005; Chu et al. 2006; Pereira et al. 2006; Tureyen et al. 2007; Zhao et al. 2009; Kim et al. 2011), NMDA-induced cytotoxicity (Zhao et al. 2006, 2007), and Parkinson’s disease (Schintu et al. 2009; Ridder and Schwaninger 2012).

In our study, by the use of PPAR-γ antagonist GW9662, we demonstrated that co-treatment with GW9662 potentiated the neurotoxic effects of TBBPA and only partially restored the TBBPA-mediated decrease in the PPAR-γ protein expression. Treatment with PPAR-γ antagonist GW9662 did not influence control levels of any studied parameter. These data point to specific antagonistic action of GW9662 on PPAR-γ protein level, but suggest non-specific action of GW9662 on TBBPA-induced neurotoxicity. Cytotoxic effects of PPAR-γ antagonist were also observed by Bishop-Bailey et al. (2000) in ECV403 cells treated with bisphenol A derivative, BADGE. Interestingly, the authors demonstrated increased transcriptional activity of PPAR-γ in BADGE-treated cells. This result might be related to the partial restoration of PPAR-γ protein level in TBBPA and GW9662-treated neocortical cells, as observed in our study. There are controversies about antagonistic/agonistic potency of PPAR-γ antagonists. Most of available data showed that PPAR-γ antagonists (BADGE, GW9662) reversed the neuroprotective effects of PPAR-γ agonists, e.g., in SK-N-SH neuronal cells exposed to ischemia, in animal model of Parkinson’s disease (Kim et al. 2011; Garrido-Gil et al. 2012; Zeng et al. 2012). In contrast, BADGE exhibited agonistic properties when it was applied to the cells non-treated with PPAR-γ agonist (Bishop-Bailey et al. 2000). In our study, we used GW9662 which is known to act as irreversible PPAR-γ antagonist. We demonstrated that GW9662 did not antagonize, but potentiated toxic effects of TBBPA in neocortical cells. This effect point to non-PPAR-γ-specific effects of GW9662. Accordingly, non-PPAR-γ-specific effects of GW9662 were also reported in breast cancer and colorectal carcinoma cells (Seargent et al. 2004; Schaefer et al. 2007).

We hypothesize, that in our study paradigm, TBBPA exhibited properties of PPAR-γ inverse agonist, i.e., it inhibited constitutive activity of the receptor. Our study demonstrated that TBBPA caused substantial decrease in PPAR-γ protein expression, much below the control level, which supports our hypothesis. Co-treatment of TBBPA with PPAR-γ agonist GW1929 inhibited the TBBPA-induced effect and normalized PPAR-γ protein level, but not above the control level. It is possible that both TBBPA and GW1929 competed for the same binding site on the receptor, thus the effect of PPAR-γ agonist GW1929 was compromised by PPAR-γ inverse agonist TPPBA. Furthermore, co-treatment of TBBPA with PPAR-γ agonist GW1929 inhibited the TBBPA-induced apoptosis and toxicity. Less conclusive were data on the effects of co-treatment of TBBPA with PPAR-γ antagonist GW9662. We demonstrated that GW9662 antagonized the TBBPA-induced decrease in PPAR-γ protein level, but it did not antagonized the TBBPA-induced apoptosis and cytotoxicity. These effects suggest that GW9662 may not be specific PPAR-γ antagonist in neuronal cells. Moreover, one may assume that TBBPA action on neuronal cells is only partially mediated by PPAR-γ, thus its actions may only be partially affected by PPAR-γ antagonist.

## Conclusion

In conclusion, our results provide a line of evidence indicating that PPAR-γ is involved in the mechanism of TBBPA action in neuronal cells. TBBPA-induced caspase-3 activity, apoptotic body formation and LDH release in neuronal cells and reduced the PPAR-γ protein expression below the control level. TBBPA-induced effects were inhibited by the PPAR-γ agonist GW1929. The PPAR-γ antagonist GW9662 prevented the TBBPA-induced decrease in the PPAR-γ protein expression. However, it potentiated TBBPA-induced apoptotic and neurotoxic effects, which suggest that the mechanism of TBBPA action in neuronal cells is not only PPAR-γ-dependent. Therefore, further studies of the mechanism of TBBPA action in the nervous system are needed.

## References

[CR1] Akasaki Y, Liu G, Matundan HH, Ng H, Yuan X, Zeng Z, Black KL, Yu JS (2006). A peroxisome proliferator-activated receptor-gamma agonist, troglitazone, facilitates caspase-8 and -9 activities by increasing the enzymatic activity of protein-tyrosine phosphatase-1B on human glioma cells. J Biol Chem.

[CR2] Alaee M, Arias P, Sjödin A, Bergman A (2003). An overview of commercially used brominated flame retardants, their applications, their use patterns in different countries/regions and possible modes of release. Environ Int.

[CR3] Al-Mousa F, Michelangeli F (2012). Some commonly used brominated flame retardants cause Ca2^+^-ATPase inhibition, beta-amyloid peptide release and apoptosis in SH-SY5Y neuronal cells. PLoS ONE.

[CR4] Birnbaum LS, Staskal DF (2004). Brominated flame retardants: cause for concern?. Environ Health Perspect.

[CR5] Bishop-Bailey D, Hla T, Warner TD (2000). Bisphenol A diglycidyl ether (BADGE) is a PPAR-γ agonist in an ECV304 cell line. Br J Pharmacol.

[CR6] Brewer GJ (1995). Serum-free B27/neurobasal medium supports differentiated growth of neurons from the striatum, substantia nigra, septum, cerebral cortex, cerebellum, and dentate gyrus. J Neurosci Res.

[CR7] Chiang MC, Cheng YC, Lin KH, Yen CH (2013). PPAR-γ regulates the mitochondrial dysfunction in human neural stem cells with tumor necrosis factor alpha. Neuroscience.

[CR8] Chu K, Lee ST, Koo JS, Jung KH, Kim EH, Sinn DI, Kim JM, Ko SY, Kim SJ, Song EC, Kim M, Roh JK (2006). Peroxisome proliferator-activated receptor-gamma-agonist, rosiglitazone, promotes angiogenesis after focal cerebral ischemia. Brain Res.

[CR9] Cimini A, Benedetti E, Cristiano L, Sebastiani P, D’Amico MA, D’Angelo B, Di Loreto S (2005). Expression of peroxisome proliferator-activated receptors (PPARs) and retinoic acidreceptors (RXRs) in rat cortical neurons. Neuroscience.

[CR10] Covaci A, Voorspoels S, Abdallah MA, Geens T, Harrad S, Law RJ (2009). Analytical and environmental aspects of the flame retardant tetrabromobisphenol-A and its derivatives. J Chromatogr A.

[CR11] Cristiano L, Bernardo A, Cerù MP (2001). Peroxisome proliferator-activated receptors (PPARs) and peroxisomes in rat cortical and cerebellar astrocytes. J Neurocytol.

[CR12] de Wit CA, Herzke D, Vorkamp K (2010). Brominated flame retardants in the Arctic environment–trends and new candidates. Sci Total Environ.

[CR13] Garrido-Gil P, Joglar B, Rodriguez-Perez AI, Guerra MJ, Labandeira-Garcia JL (2012). Involvement of PPAR-γ in the neuroprotective and anti-inflammatory effects of angiotensin type 1 receptor inhibition: effects of the receptor antagonist telmisartan and receptor deletion in a mouse MPTP model of Parkinson’s disease. J Neuroinflammation.

[CR14] Gray E, Ginty M, Kemp K, Scolding N, Wilkins A (2012). The PPAR-γ agonist pioglitazone protects cortical neurons from inflammatory mediators via improvement in peroxisomal function. J Neuroinflammation.

[CR15] Hendriks HS, van Kleef RG, van den Berg M, Westerink RH (2012). Multiple novel modes of action involved in the in vitro neurotoxic effects of tetrabromobisphenol-A. Toxicol Sci.

[CR16] Heneka MT, Klockgether T, Feinstein DL (2000). Peroxisome proliferator-activated receptor-gamma ligands reduce neuronal inducible nitric oxide synthase expression and cell death in vivo. J Neurosci.

[CR17] Johnson-Restrepo B, Adams DH, Kannan K (2008). Tetrabromobisphenol A (TBBPA) and hexabromocyclododecanes (HBCDs) in tissues of humans, dolphins, and sharks from the United States. Chemosphere.

[CR18] Kajta M, Lasoń W, Kupiec T (2004). Effects of estrone on N-methyl-d-aspartic acid- and staurosporine-induced changes in caspase-3-like protease activity and lactate dehydrogenase-release: time- and tissue-dependent effects in neuronal primary cultures. Neuroscience.

[CR19] Kajta M, Trotter A, Lasoń W, Beyer C (2005). Effect of NMDA on staurosporine-induced activation of caspase-3 and LDH release in mouse neocortical and hippocampal cells. Brain Res Dev Brain Res.

[CR20] Kaundal RK, Sharma SS (2011). Ameliorative effects of GW1929, a nonthiazolidinedione PPAR-γ agonist, on inflammation and apoptosis in focal cerebral ischemic-reperfusion injury. Curr Neurovasc Res.

[CR21] Kibakaya EC, Stephen K, Whalen MM (2009). Tetrabromobisphenol A has immunosuppressive effects on human natural killer cells. J Immunotoxicol.

[CR22] Kiciński M, Viaene MK, Den Hond E, Schoeters G, Covaci A, Dirtu AC, Nelen V, Bruckers L, Croes K, Sioen I, Baeyens W, Van Larebeke N, Nawrot TS (2012). Neurobehavioral function and low-level exposure to brominated flame retardants in adolescents: a cross-sectional study. Environ Health.

[CR23] Kim EJ, Park KS, Chung SY, Sheen YY, Moon DC, Song YS, Kim KS, Song S, Yun YP, Lee MK, Oh KW, Yoon DY, Hong JT (2003). Peroxisome proliferator-activated receptor-gamma activator 15-deoxy-Delta12,14-prostaglandin J2 inhibits neuroblastoma cell growth through induction of apoptosis: association with extracellular signal-regulated kinase signal pathway. J Pharmacol Exp Ther.

[CR24] Kim KY, Cho HS, Lee SH, Ahn JH, Cheon HG (2011). Neuroprotective effects of KR-62980, a new PPAR-γ agonist, against chemical ischemia-reperfusion in SK-N-SH cells. Brain Res.

[CR25] Kitamura Y, Shimohama S, Koike H, Kakimura Ji, Matsuoka Y, Nomura Y, Gebicke-Haerter PJ, Taniguchi T (1999). Increased expression of cyclooxygenases and peroxisome proliferator-activated receptor-gamma in Alzheimer’s disease brains. Biochem Biophys Res Commun.

[CR26] Moreno S, Farioli-Vecchioli S, Cerù MP (2004). Immunolocalization of peroxisome proliferator-activated receptors and retinoid X receptors in the adult rat CNS. Neuroscience.

[CR27] Nakajima A, Saigusa D, Tetsu N, Yamakuni T, Tomioka Y, Hishinuma T (2009). Neurobehavioral effects of tetrabromobisphenol A, a brominated flame retardant, in mice. Toxicol Lett.

[CR28] Nicholson DW, Ali A, Thornberry NA, Vaillancourt JP, Ding CK, Gallant M, Gareau Y, Griffin PR, Labelle M, Lazebnik YA (1995). Identification and inhibition of the ICE/CED-3 protease necessary for mammalian apoptosis. Nature.

[CR29] Pereira MP, Hurtado O, Cárdenas A, Boscá L, Castillo J, Dávalos A, Vivancos J, Serena J, Lorenzo P, Lizasoain I, Moro MA (2006). Rosiglitazone and 15-deoxy-Delta12,14-prostaglandin J2 cause potent neuroprotection after experimental stroke through noncompletely overlapping mechanisms. J Cereb Blood Flow Metab.

[CR30] Reistad T, Mariussen E, Ring A, Fonnum F (2007). In Vitro Toxicity of Tetrabromobisphenol-A on Cerebellar Granule Cells: cell Death. Free Radical Formation, Calcium Influx and Extracellular Glutamate, Toxicological Sciences.

[CR31] Ridder DA, Schwaninger M (2012). In search of the neuroprotective mechanism of thiazolidinediones in Parkinson’s disease. Exp Neurol.

[CR32] Riu A, Grimaldi M, le Maire A, Bey G, Phillips K, Boulahtouf A, Perdu E, Zalko D, Bourguet W, Balaguer P (2011). Peroxisome proliferator-activated receptor γ is a target for halogenated analogs of bisphenol A. Environ Health Perspect.

[CR33] Riu A, le Maire A, Grimaldi M, Audebert M, Hillenweck A, Bourguet W, Balaguer P, Zalko D (2011). Characterization of novel ligands of ERα, Erβ, and PPAR-γ: the case of halogenated bisphenol A and their conjugated metabolites. Toxicol Sci.

[CR34] Schaefer KL, Takahashi H, Morales VM, Harris G, Barton S, Osawa E, Nakajima A, Saubermann LJ (2007). PPAR-γ Inhibitors Reduce Tubulin Protein Levels by a PPARgamma, PPARdelta and Proteasome-Independent Mechanism, Resulting in Cell Cycle Arrest, Apoptosis and Reduced Metastasis of Colorectal Carcinoma Cells. Int J Cancer.

[CR35] Schauer UM, Völkel W, Dekant W (2006). Toxicokinetics of tetrabromobisphenol a in humans and rats after oral administration. Toxicol Sci.

[CR36] Schintu N, Frau L, Ibba M, Garau A, Carboni E, Carta AR (2009). Progressive dopaminergic degeneration in the chronic MPTPp mouse model of Parkinson’s disease. Neurotox Res.

[CR37] Seargent JM, Yates EA, Gill JH (2004). GW9662, a potent antagonist of PPAR-γ, inhibits growth of breast tumour cells and promotes the anticancer effects of the PPAR-γ agonist rosiglitazone, independently of PPARgamma activation. Br J Pharmacol.

[CR38] Sellstrom U, Jansson B (1995). Analysis of tetrabromobisphenol a in a product and environmental-samples. Chemosphere.

[CR39] Shi H, Qian L, Guo S, Zhang X, Liu J, Cao Q (2010). Teratogenic effects of tetrabromobisphenol A on Xenopus tropicalis embryos. Comp Biochem Physiol C.

[CR40] Shimazu T, Inoue I, Araki N, Asano Y, Sawada M, Furuya D, Nagoya H, Greenberg JH (2005). A peroxisome proliferator-activated receptor-gamma agonist reduces infarct size in transient but not in permanent ischemia. Stroke.

[CR41] Sjödin A, Patterson DG, Bergman A (2003). A review on human exposure to brominated flame retardants–particularly polybrominated diphenyl ethers. Environ Int.

[CR42] Talsness CE, Andrade AJ, Kuriyama SN, Taylor JA, vom Saal FS (2009). Components of plastic: experimental studies in animals and relevance for human health. Philos Trans R Soc Lond B Biol Sci.

[CR43] Thomsen C, Lundanes E, Becher G (2002). Brominated flame retardants in archived serum samples from Norway: a study on temporal trends and the role of age. Environ Sci Technol.

[CR44] Tureyen K, Kapadia R, Bowen KK, Satriotomo I, Liang J, Feinstein DL, Vemuganti R (2007). Peroxisome proliferator-activated receptor-gamma agonists induce neuroprotection following transient focal ischemia in normotensive, normoglycemic as well as hypertensive and type-2 diabetic rodents. J Neurochem.

[CR45] Van der Ven LT, Van de Kuil T, Verhoef A, Verwer CM, Lilienthal H, Leonards PE, Schauer UM, Cantón RF, Litens S, De Jong FH, Visser TJ, Dekant W, Stern N, Håkansson H, Slob W, Van den Berg M, Vos JG, Piersma AH (2008). Endocrine effects of tetrabromobisphenol-A (TBBPA) in Wistar rats as tested in a one-generation reproduction study and a subacute toxicity study. Toxicology.

[CR46] Viberg H, Eriksson P (2011). Differences in neonatal neurotoxicity of brominated flame retardants, PBDE 99 and TBBPA, in mice. Toxicology.

[CR47] Wada K, Nakajima A, Katayama K, Kudo C, Shibuya A, Kubota N, Terauchi Y, Tachibana M, Miyoshi H, Kamisaki Y, Mayumi T, Kadowaki T, Blumberg RS (2006). Peroxisome proliferator-activated receptor gamma-mediated regulation of neural stem cell proliferation and differentiation. J Biol Chem.

[CR48] Yi KD, Covey DF, Simpkins JW (2009). Mechanism of okadaic acid-induced neuronal death and the effect of estrogens. J Neurochem.

[CR49] Zeng Y, Xie K, Dong H, Zhang H, Wang F, Li Y, Xiong L (2012). Hyperbaric oxygen preconditioning protects cortical neurons against oxygen-glucose deprivation injury: role of peroxisome proliferator-activated receptor-gamma. Brain Res.

[CR50] Zhao X, Ou Z, Grotta JC, Waxham N, Aronowski J (2006). Peroxisome-proliferator-activated receptor-gamma (PPARgamma) activation protects neurons from NMDA excitotoxicity. Brain Res.

[CR51] Zhao X, Sun G, Zhang J, Strong R, Song W, Gonzales N, Grotta JC, Aronowski J (2007). Hematoma resolution as a target for intracerebral hemorrhage treatment: role for peroxisome proliferator-activated receptor gamma in microglia/macrophages. Ann Neurol.

[CR52] Zhao X, Strong R, Zhang J, Sun G, Tsien JZ, Cui Z, Grotta JC, Aronowski J (2009). Neuronal PPARgamma deficiency increases susceptibility to brain damage after cerebral ischemia. J Neurosci.

[CR53] Zhao Y, Calon F, Julien C, Winkler JW, Petasis NA, Lukiw WJ, Bazan NG (2011). Docosahexaenoic acid-derived neuroprotectin D1 induces neuronal survival via secretase- and PPARγ-mediated mechanisms in Alzheimer’s disease models. PLoS ONE.

